# Evaluation of 3D multi-contrast joint intra- and extracranial vessel wall cardiovascular magnetic resonance

**DOI:** 10.1186/s12968-015-0143-z

**Published:** 2015-05-27

**Authors:** Zechen Zhou, Rui Li, Xihai Zhao, Le He, Xiaole Wang, Jinnan Wang, Niranjan Balu, Chun Yuan

**Affiliations:** Center for Biomedical Imaging Research, Department of Biomedical Engineering, School of Medicine, Tsinghua University, Beijing, China; Department of Biomedical Engineering, Tsinghua University, Beijing, China; Department of Radiology, University of Washington, Seattle, WA USA; Philips Research North America, Briarcliff Manor, NY USA

**Keywords:** Atherosclerosis, Vessel wall imaging, Carotid artery, Intracranial artery, 3D-MERGE, SNAP, VISTA, Magnetic resonance

## Abstract

**Background:**

Multi-contrast vessel wall cardiovascular magnetic resonance (CMR) has demonstrated its capability for atherosclerotic plaque morphology measurement and component characterization in different vasculatures. However, limited coverage and partial volume effect with conventional two-dimensional (2D) techniques might cause lesion underestimation. The aim of this work is to evaluate the performance in a) blood suppression and b) vessel wall delineation of three-dimensional (3D) multi-contrast joint intra- and extracranial vessel wall imaging at 3T.

**Methods:**

Three multi-contrast 3D black blood (BB) sequences with T1, T2 and heavy T1 weighting and a custom designed 36-channel neurovascular coil covering the entire intra- and extracranial vasculature have been used and investigated in this study. Two healthy subjects were recruited for sequence parameter optimization and twenty-five patients were consecutively scanned for image quality and blood suppression assessment. Qualitative image scores of vessel wall delineation as well as quantitative Signal-to-Noise Ratio (SNR) and Contrast-to-Noise Ratio (CNR) were evaluated at five typical locations ranging from common carotid arteries to middle cerebral arteries.

**Results:**

The 3D multi-contrast images acquired within 15mins allowed the vessel wall visualization with 0.8 mm isotropic spatial resolution covering intra- and extracranial segments. Quantitative wall and lumen SNR measurements for each sequence showed effective blood suppression at all selected locations (P < 0.0001). Although the wall-lumen CNR varied across measured locations, each sequence provided good or adequate image quality in both intra- and extracranial segments.

**Conclusions:**

The proposed 3D multi-contrast vessel wall technique provides isotropic resolution and time efficient solution for joint intra- and extracranial vessel wall CMR.

## Background

Atherosclerosis is a major cause of ischemic stroke and can develop in multiple vascular beds [1, 2]. The presence of concurrent intra- and extracranial atherosclerosis is found to be prevalent in stroke patients [3]. In recent years, BB Vessel Wall Imaging (VWI) technique using cardiovascular magnetic resonance (CMR) has been validated to be an effective method for characterizing atherosclerotic plaque and demonstrated its potential for risk stratification. Clinically, the most widely used VWI technique is 2D multi-contrast BB sequences [4, 5]. The multi-contrast approach ensures the definition of vessel luminal surface condition, plaque distribution, and plaque burden as well as the detection of key plaque components [6–8]. But the 2D approach is limited in longitudinal coverage and partial volume effect caused by long acquisition time and large slice thickness respectively. This can be further exacerbated by the tortuosity of blood vessels particularly for intracranial arteries.

Recently, several high resolution 3D isotropic BB sequences at 3 T have been proposed to improve the accuracy of plaque burden measurement and plaque components detection for either intracranial VWI [9, 10] or extracranial carotid VWI [11–13] in comparison to 2D imaging. In addition, previous studies using 2D multi-contrast carotid CMR have identified that a basic set of sequences including T1, T2 and heavy T1 weightings can be used for plaque imaging [14–16]. Since 3D Motion Sensitized Driven Equilibrium (MSDE) prepared Rapid Gradient Echo (3D-MERGE), T2-weighted Volumetric ISotropic Turbo spin echo Acquisition (VISTA), and Simultaneous Noncontrast Angiography and intraPlaque hemorrhage (SNAP) together meet this multi-contrast requirement, they have the potential to be optimized and applied for joint intra- and extracranial screening of atherosclerosis.

However, to jointly delineate intra- and extracranial vessel wall using 3D multi-contrast sequences, several challenges need to be addressed. a) Imaging coverage and overall scan time. Simply utilizing multi-station or dual stack [17] strategy may cause misregistration between stations or stacks and lead to long scan times. b) Effective blood suppression in a large region where various blood flow pattern, direction, and velocities co-exist must be considered by specific blood suppression techniques. c) Image quality of vessel wall delineation must be adequate for clinical diagnosis of plaque. Both SNR and resolution challenges must be optimized for vessel wall boundary and plaque components depiction across multiple arteries.

In this study, we optimized 3D-MERGE, T2-weighted VISTA and SNAP into a 3D multi-contrast protocol combined with the advantage of a higher SNR and larger coverage neurovascular coil [18] to achieve a time efficient solution for joint intra- and extracranial VWI. The performance of this joint 3D plaque CMR protocol was investigated on stroke patients to test blood suppression effectiveness and vessel wall delineation ranging from the extracranial carotid to intracranial arteries.

## Methods

### Study population

In this Institutional Review Board approved study, 2 healthy volunteers (26 year-old males) were recruited to optimize sequence parameters for blood suppression, SNR, and wall delineation. Afterwards, 25 patients (17 males, age: 26–79 years, median: 55 years) with recent (within 2 weeks) ischemic stroke or Transient Ischemic Attack (TIA) were consecutively recruited after informed consent.

### Sequence optimization

**Fig. 1:**
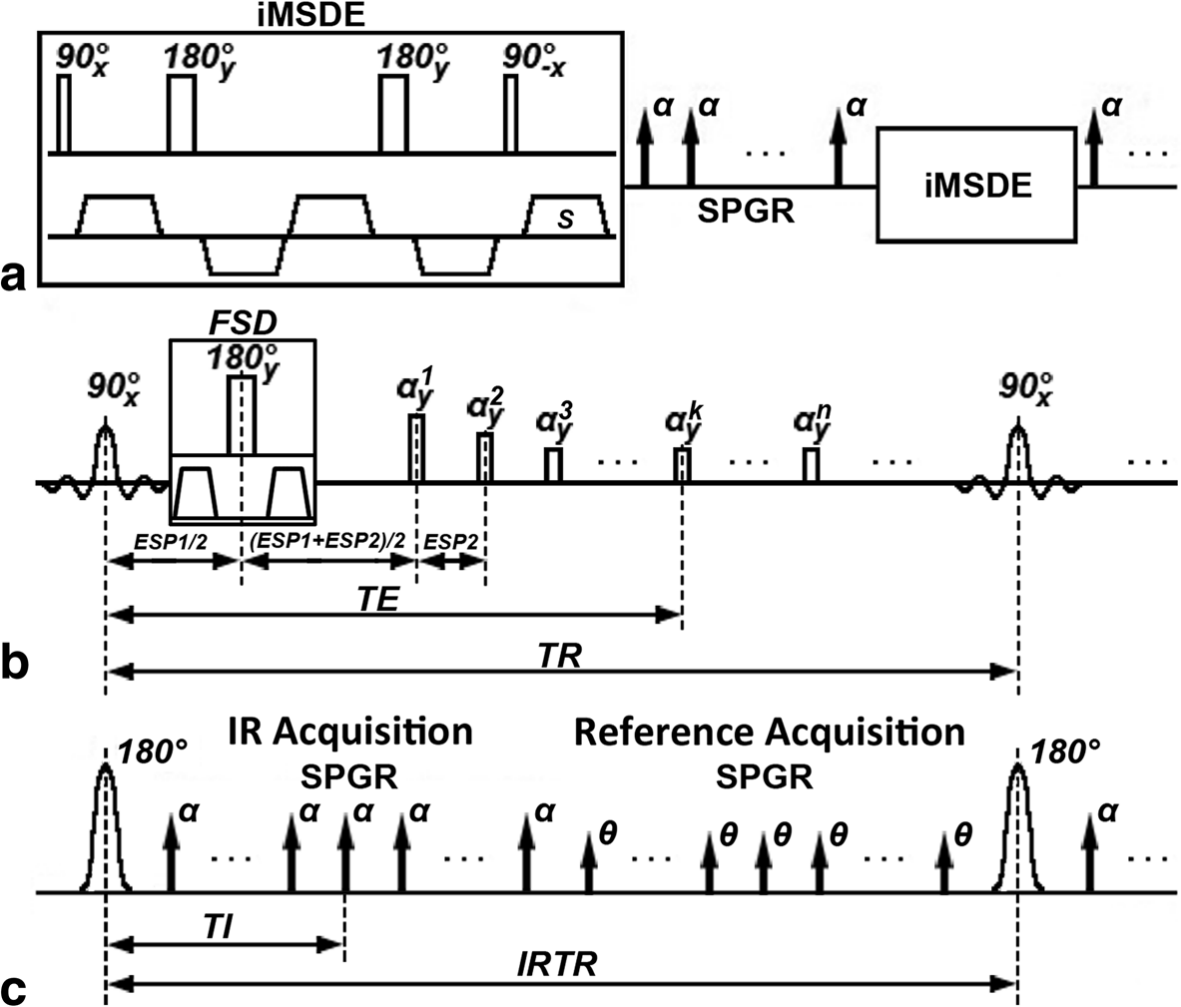
Sequence diagram of 3D-MERGE, T2-weighted VISTA and SNAP. (**a** - **c**) corresponds respectively to the pulse sequence diagram of 3D-MERGE, T2-weighted VISTA and SNAP. The fat saturation scheme used both for 3D-MERGE and T2-weighted VISTA is spectral presaturation inversion recovery (SPIR) and for SNAP is water selective excitation in practice but they are not shown in this schematic figure

The improved MSDE (iMSDE) [19] preparation in 3D-MERGE (Fig. 1(a)) was implemented along all three orthogonal directions. A cumulative first order gradient moment (M_1_) of 500 mT*ms^2^/m, 1000 mT*ms^2^/m and 1500 mT*ms^2^/m were evaluated for vessel wall and lumen SNR measured in both intra- and extracranial segments during healthy volunteer scans, where 20 cross sectional slices were extracted with equal interval along both sides of the intra- and extracranial vasculatures. The preparation duration of iMSDE was minimized with the maximum gradient strength of 20 mT/m to reduce the signal loss caused by T2 decay or rephasing error induced by field inhomogeneity, eddy current and motion. After iMSDE preparation, a train of SPoiled GRadient echo (SPGR) with centric ordering was used for k-space data acquisition in 3D-MERGE [11].

For T2-weighted VISTA (Fig. 1(b)), FSD gradients were applied along the readout direction to efficiently suppress the excited blood spins within the imaging volume. After establishment of Static Pseudo Steady State (SPSS), the hard refocusing RF pulses were performed with Reduced Flip Angle (RFA) scheme. The refocusing flip angle and the echo train length were optimized using Extended Phase Graph (EPG) [20–23] in terms of SNR efficiency, which defined as a product of central echo intensity and a ratio between acquisition duration in each shot and the shot duration/repetition time. In this simulation, the repetition time was fixed to 2500 ms and the T1 and T2 relaxation times of 1114 ms and 55 ms respectively [24] were used as simulation parameters for vessel wall. The optimized RFA scheme was further compared with Variable Flip Angle (VFA) scheme in healthy volunteers with the identical effective echo time and repetition time to evaluate the quality for vessel wall delineation.

For SNAP (Fig. 1(c)), both Inversion Recovery (IR) and reference acquisition were acquired in linear ordering to guarantee the signal polarity requirement and minimize the background phase difference between IR and reference images [25]. A B1 insensitive nonselective adiabatic inversion pulse was applied and the inversion time (TI) was selected to meet the tissue signal polarity requirement and blood outflow condition [26]. In volunteer study, the nonselective inversion RF pulse was compared with its selective counterpart to investigate the suppression performance for blood inflow artifacts.

### CMR

**Fig. 2:**
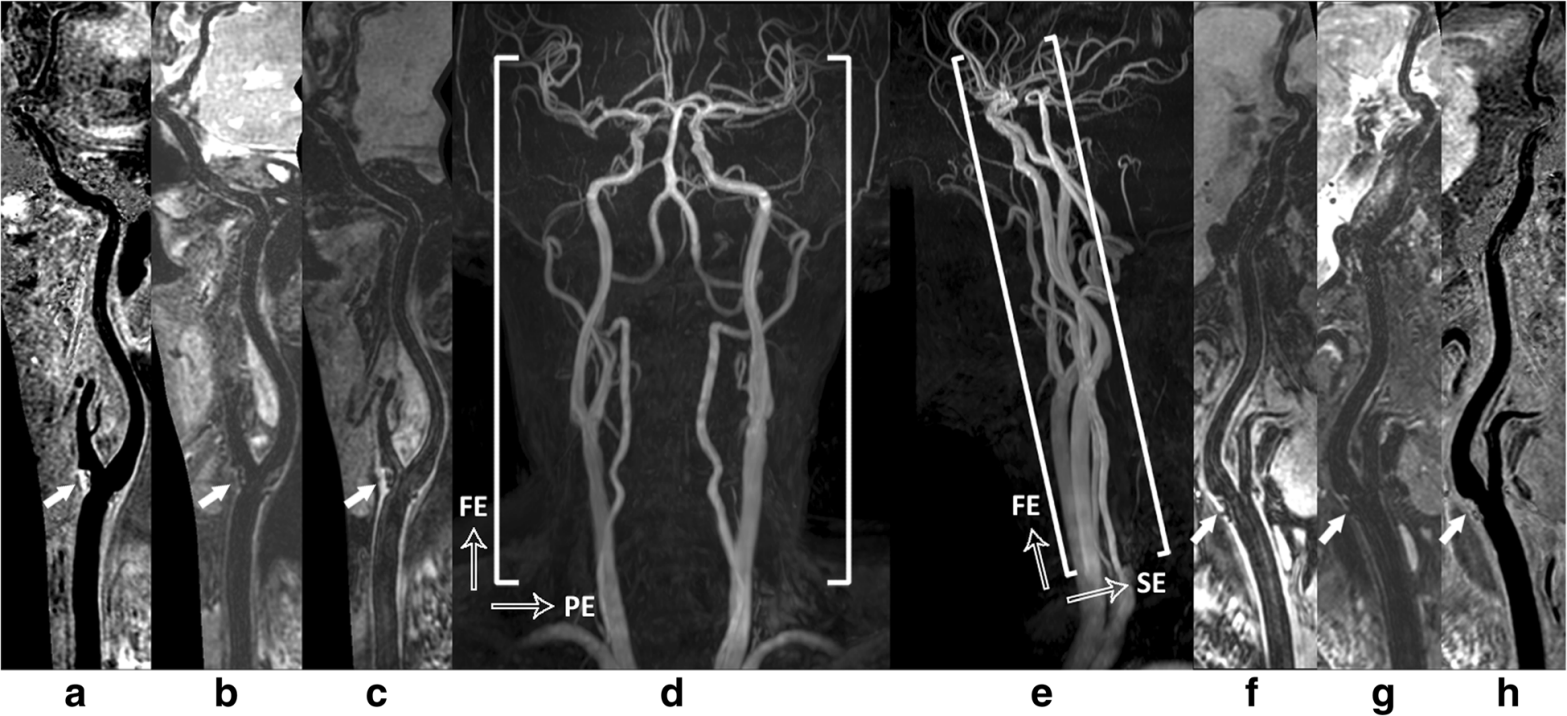
Illustration of 3D multi-contrast imaging coverage. Three stations of 3D time-of-flight coronal maximum intensity projection (MIP) fusion (**d**) and sagittal MIP fusion (**e**) are illustrated here only for a clear definition of imaging FOV. The curved multi-planar reconstruction (MPR) examples show the right (**a** - **c**) and left (**f** - **h**) sides arteries ranging from CCA through ICA to MCA, where (**c**, **f**), (**b**, **g**), and (**a**, **h**) correspond to the results of 3D-MERGE, T2-weighted VISTA and SNAP respectively. Note the plaques were detected at bilateral carotid bifurcations on all different contrast weighted images as shown by solid arrows

To support VWI across multiple vasculatures, receiving coil had to provide sufficient longitudinal coverage and SNR performance. In this work, a recently developed 36-channel neurovascular coil was employed with improved SNR performance at both intra- and extracranial segments in comparison to the commercialized 8-channel carotid/head coil and 16-channel neurovascular coil [18]. The head and carotid elements of this 36-channel coil were activated as MR signal receivers. With this large coverage coil configuration, a single 3D Cartesian slab (Fig. 2) can be prescribed to cover at least Common Carotid Arteries (CCA), Internal Carotid Arteries (ICA) and Middle Cerebral Arteries (MCA). This extended longitudinal FOV along Foot-Head (FH) direction can cover the most common sites for intra- and extracranial atherosclerosis [2] and avoid the potential imaging gaps or prolonged preparation time due to switching between carotid and head coils. Also, the overall scan time can be optimally controlled due to less number of phase encodes in Left-Right (LR) direction and thin slab thickness along Anterior-Posterior (AP) direction.

All experiments were performed on a 3 T MR scanner (Philips Achieva TX, Best, Netherland). All subjects were instructed to avoid swallowing during scans. Following 3-plane survey, a fast large coverage 2D-PCA sequence was obtained for vessel localization (41 s). Then three coronal acquisitions of 3D-MERGE, T2-weighted VISTA and SNAP were performed with identical scan plane and same spatial resolution covering CCA, ICA and MCA. To maximize the available SNR and image quality, imaging acceleration methods were not considered in this study. The 3D multi-contrast protocol used herein (Table 1) can achieve isotropic 0.8 mm resolution to cover the major intracranial and entire carotid arteries within 15mins.

**Table 1:** Imaging parameters for 3D multi-contrast sequences

	3D-MERGE	T2-weighted VISTA	SNAP
FOV (FHxLRxAP mm^3^)	250x160x40		
Resolution (FHxLRxAP mm^3^) for acquisition/reconstruction	0.8x0.8x0.8/0.4x0.4x0.4		
TE (ms)	4.2	255/86^a^	4.5
TR (ms)	9	2500	10/1987^b^
Echo spacing (ms)	NA	10.8/3.6^c^	NA
Flip angle (deg)	6	32	11/5^d^
Turbo factor	90	130 + 4^e^	98
Averages	2	1	2
Scan time (min)	3:10	4:32	6:47

^a^Effective echo time/Equivalent echo time

^b^SPGR repetition time/inversion recovery repetition time (IRTR)

^c^Echo spacing for the first/other refocusing interval

^d^Flip angle used in IR/reference acquisition

^e^Number of echoes used in each shot for acquisition/SPSS start-up

### Image analysis

Both qualitative and quantitative analysis were performed at five locations, including CCA, carotid bifurcation (BULB), ICA C2, ICA C5 and MCA M1 segment [27, 28]. Each location was selected at the region with complex flow and various surrounding tissues, which is difficult for blood suppression and out wall boundary delineation. A curved MPR was firstly obtained on each 3D isotropic image dataset using open-source software Osirix [29]. Then 0.5 mm thick 2D cross-sectional slices were obtained at all five bilateral locations. The following qualitative and quantitative analysis were both conducted on these reconstructed 2D images.

Qualitative analysis was used to evaluate image quality on vessel wall delineation at each location for each sequence. All images were independently reviewed by two observers both with four-year experience in VWI. Image quality was scored using a four-level criteria. Score level from 4 to 1 corresponded to excellent, good, adequate and inadequate image quality. More specifically, excellent rated images showed clear vessel wall delineation for entire boundary. Good MR images represented good vessel wall delineation with only small part of obscure/invisible boundary. Adequate rated images stood for reasonable image quality on vessel wall visualization involving some part but less than a quadrant of obscure/invisible boundary. Finally, inadequate were acquisitions in which most of the vessel wall boundaries could not be seen.

Quantitative analysis was focused on evaluation of blood suppression effectiveness at each location. SNR measurements were performed with the free software ImageJ (version 1.48, National Institutes of Health, Bethesda, MD). Lumen signal (S_l_) was measured as the mean intensity within a Region-Of-Interest (ROI) manually drawn to cover the whole arterial lumen. For SNAP, S_l_ measured on negative images were transformed into its opposite sign to avoid negative value. Arterial wall signal (S_w_) was measured as the mean signal intensity of pixels manually selected on the middle path of vessel wall boundary. The SNR of wall (SNR_w_) and lumen (SNR_l_) can be calculated as SNR_w_ = S_w_/σ_n_ and SNR_l_ = S_l_/σ_n_ respectively, where σ_n_ is the standard deviation of noise. Because of the inhomogeneous noise distribution due to spatially varying sensitivity map and post-processing image filter, σ_n_ was measured as the standard deviation within a ROI manually placed in the adjacent white matter for intracranial segment and muscle for extracranial carotid segment instead of the background air [30, 31]. The CNR between wall and lumen (CNR_w-l_) at each location were calculated as CNR_w-l_ = SNR_w_ – SNR_l_ for 3D-MERGE and T2-weighted VISTA while as CNR_w-l_ = SNR_w_ + SNR_l_ for SNAP due to opposite signal polarity of lumen and wall.

### Statistical analysis

MATLAB (R2013b, Mathworks, Natick, MA) was used to perform the statistical analysis. SNR_w_ and SNR_l_ were compared at each location of one sequence using two-sided Wilcoxon signed rank test to evaluate the blood suppression performance. In all tests, statistical significance was defined at P < 0.05 level. Results are presented as column bar with error bar or mean ± standard deviation at each location.

## Results

### Sequence optimization

**Fig. 3:**
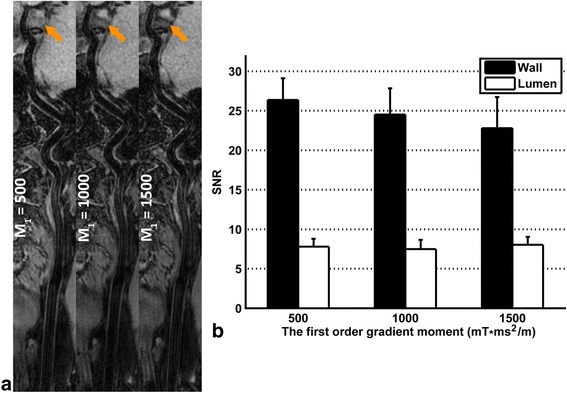
Comparison of 3D-MERGE using different first order gradient moment (M_1_) values. A representative comparison for M_1_ values ranging from 500mT*ms^2^/m to 1500mT*ms^2^/m are illustrated in (**a**). Note that CSF can be better suppressed when using larger M_1_ value (marked in yellow arrows). The vessel wall and lumen SNR are measured when different M_1_ values are used and compared in (**b**)

The volunteer study indicated that the blood can be suppressed similarly but the Cerebral Spinal Fluid (CSF) can be better suppressed with the M_1_ value increasing, as shown in Fig. 3(a), which would improve the delineation of intracranial vessel wall. Also, the preparation duration of iMSDE was longer when using larger M_1_ value so that the vessel wall SNR was penalized as shown in Fig. 3(b). With these considerations, the optimal M_1_ value was selected as 1000 mT*ms^2^/m in this study.

**Fig. 4:**
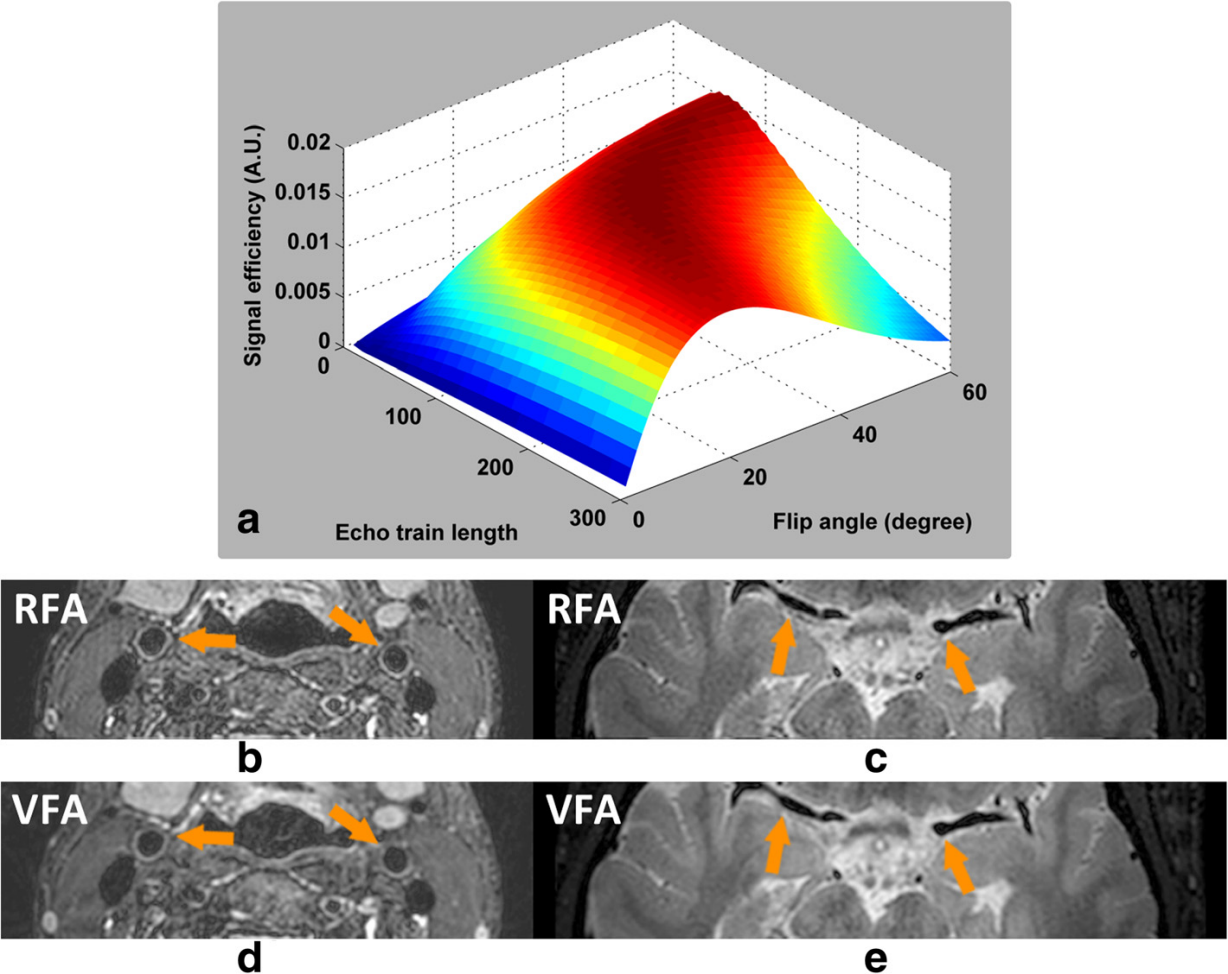
Optimization of T2-weighted VISTA echo train. Panel (**a**) shows the optimization of RFA scheme on echo train length and refocusing flip angle. The optimized RFA scheme (**b**, **c**) can further improve the sharpness of vessel wall boundary (marked in yellow arrows) but cause SNR loss compared to VFA scheme (**d**, **e**)

The EPG simulation showed that an inversely proportional relationship between refocusing flip angle and echo train length can provide a better tradeoff between the SNR of vessel wall and acquisition efficiency (Fig. 4(a)). Based on this result, a constant 32° RFA scheme with echo train length of 130 were used in this study. Also, the smaller refocusing flip angle would be beneficial for flow suppression with this relatively long echo time [32, 33]. In addition, this optimized RFA scheme can help to improve the outer wall boundary definition in comparison to VFA scheme, particularly for the intracranial arteries surrounded with CSF (Fig. 4(b-e)), because the first half high frequency k-space data acquired before the echo time was enhanced by RFA.

**Fig. 5:**
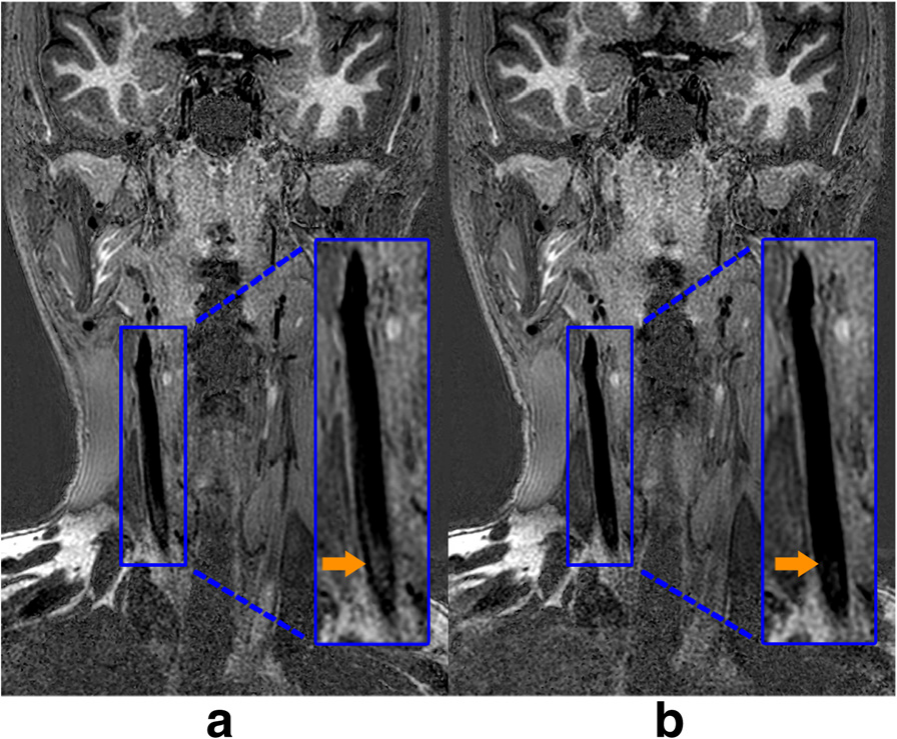
Comparison of SNAP between selective and non-selective inversion pulse. The non-selective (**b**) inversion pulse can further improve the suppression of blood inflow artifacts in comparison to its selective counterpart (**a**)

For SNAP optimization, the nonselective inversion pulse can better remove the blood inflow artifacts (Fig. 5) due to its extended inversion band and this would particularly benefit for SNAP imaging in this large coverage scenario comparing to the conventional selective inversion pulse.

### CMR

**Fig. 6:**
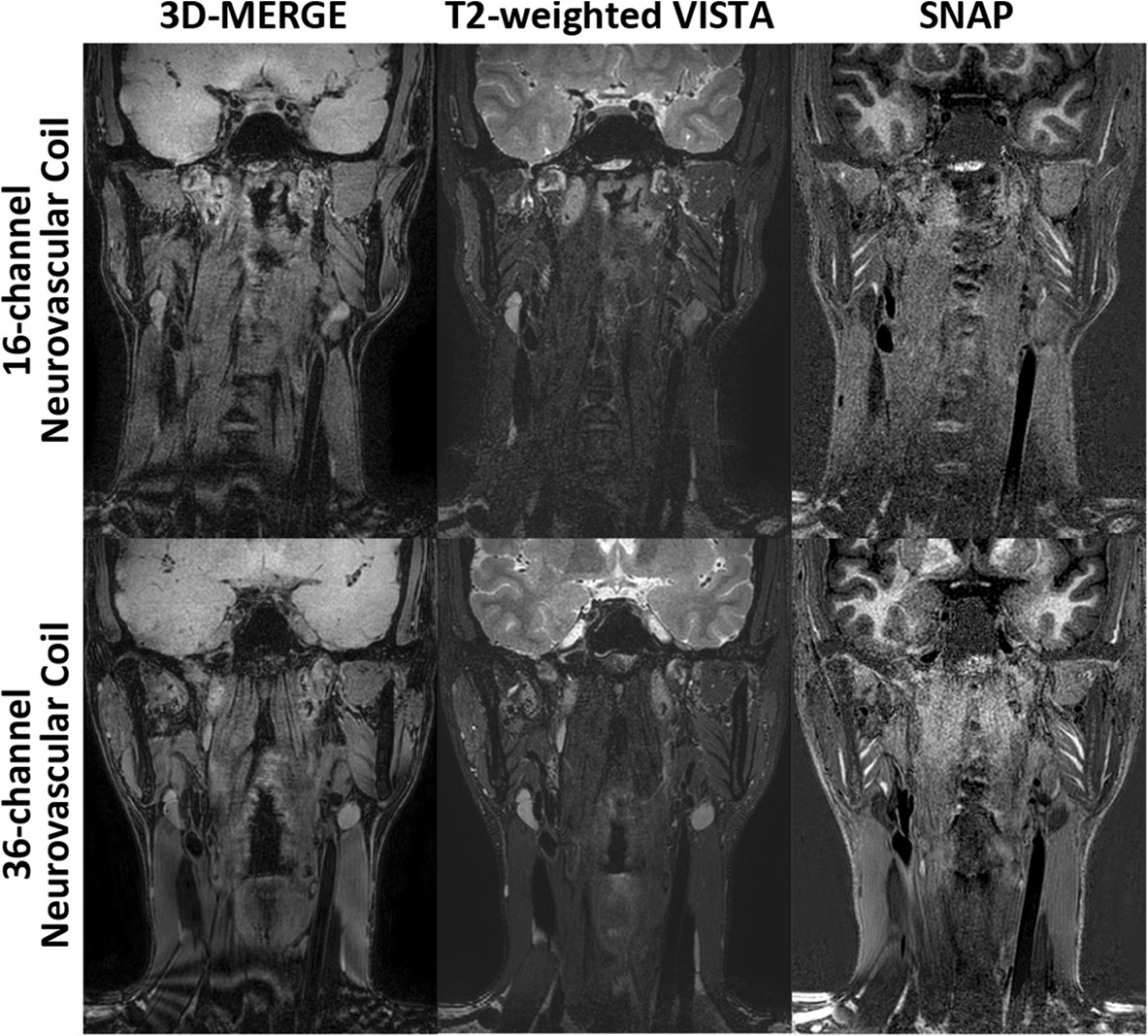
Performance comparison between different neurovascular coils. The optimized protocols are scanned using 16-channel commercialized and 36-channel developed neurovascular coil. The comparison results are shown in top and bottom row respectively

The comparison result between 16- and 36-channel neurovascular coil using the previously optimized sequence parameters further demonstrated that the developed coil can outperform the commercialized one with improved SNR particularly at extracranial segments (Fig. 6).

**Fig. 7:**
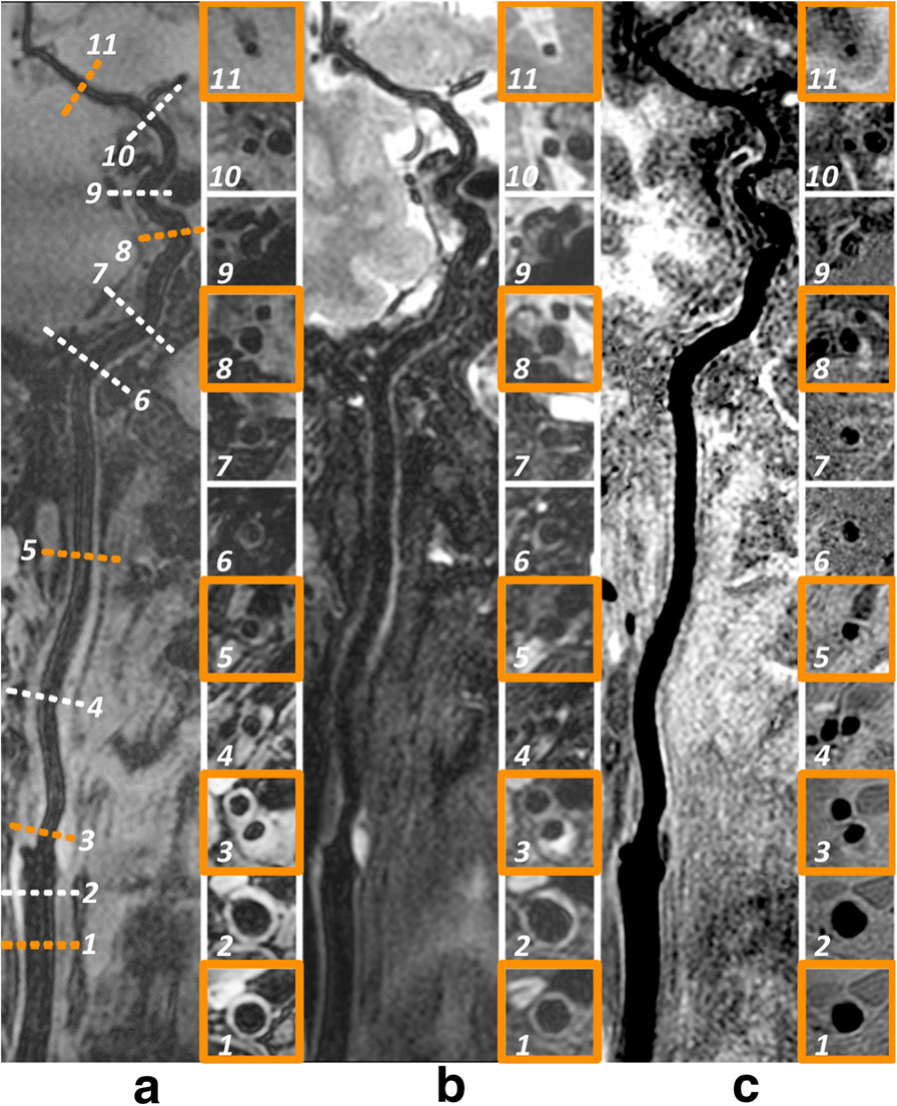
Representative blood suppression and vessel wall delineation of the proposed 3D multi-contrast imaging. The curved MPR results in (**a**-**c**) show the overall blood suppression effect from 3D-MERGE, T2-weighted VISTA and SNAP sequence. The cross-sectional views at 11 locations were also provided with numbered subwindows in (**a** - **c**), including CCA, proximal and distal carotid bifurcation, ICA C1-C7 and MCA M1. The colored 5 locations were selected for quantitative analysis in this study. Note the plaque identified at location 3 illustrates different signal behaviors on different contrast weighted 3D sequences

**Fig. 8:**
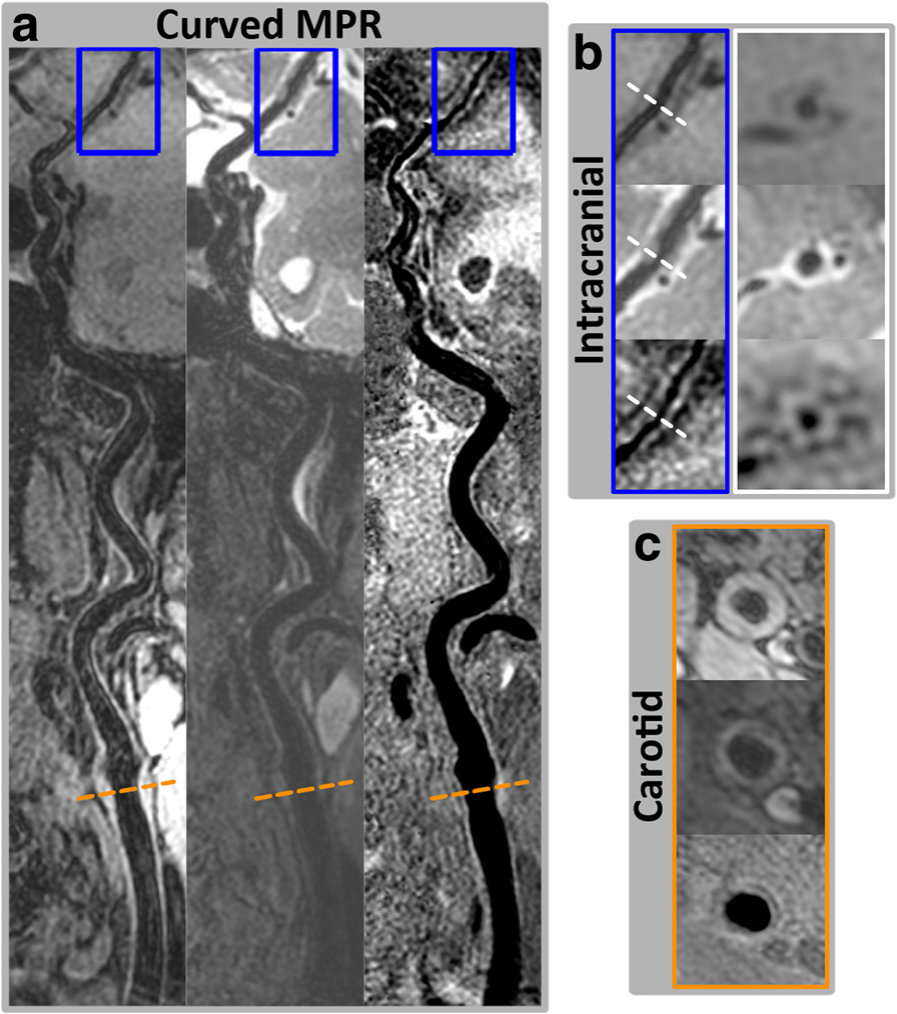
Example images showing the capability of arbitrary view for intra- and extracranial vessel wall delineation. An overview of vessel wall across multiple arteries can be provided using 3D isotropic VWI and curved MPR technique (**a**). Note that vessel wall thickening at the intracranial MCA M1 (**b**) and carotid bifurcation (**c**) can be clearly identified with 3D-MERGE, T2-weighted VISTA and SNAP sequences

**Fig. 9:**
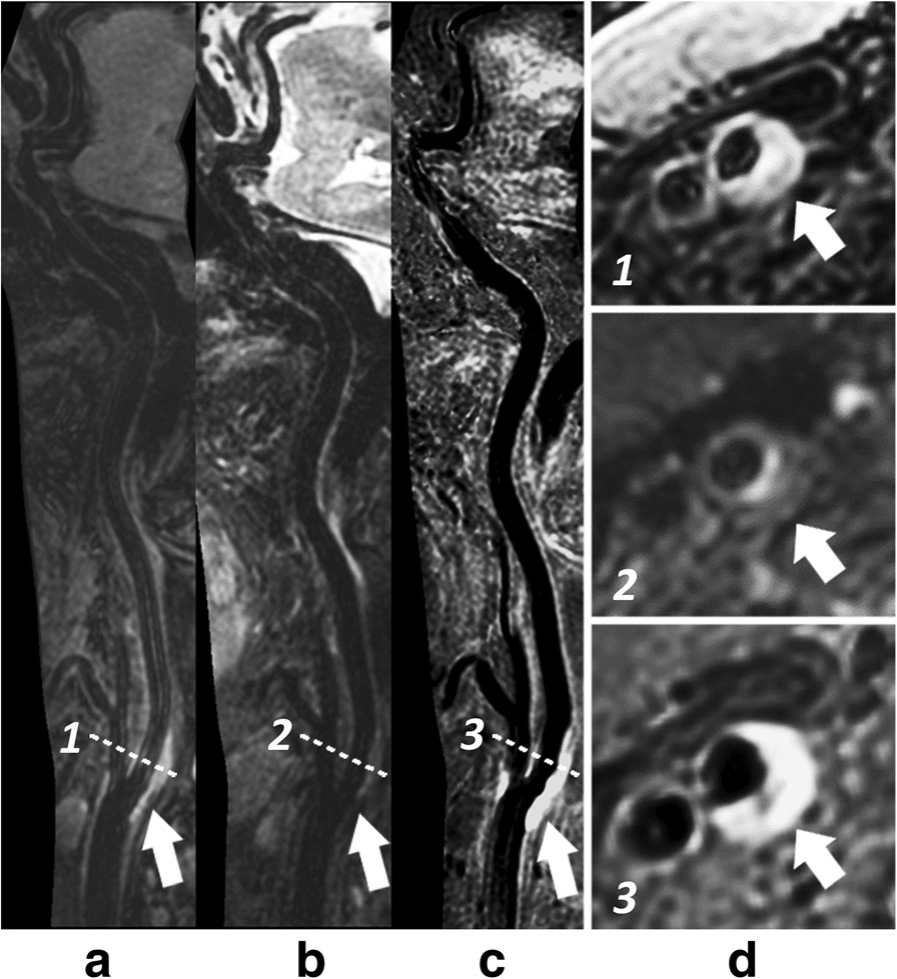
Visualization of one local plaque in 3D-MERGE, T2-weighted VISTA and SNAP images. Both curved longitudinal and cross-sectional views of the intracranial and carotid arterial walls can be observed using MPR on 3D-MERGE (**a**), T2-weighted VISTA (**b**) and SNAP (**c**) images. The locations of the 3 cross-sectional images in (**d**) are marked by the dashed lines on the curved longitudinal images. IPH component and severe wall thickening distributed around the carotid bulb can be confirmed based on the 3D multi-contrast images

The 3D multi-contrast images were successfully acquired from all participants and all datasets were used for qualitative image scoring while two subjects were excluded from quantitative image analysis since SNR measurement cannot be performed at several locations due to occlusion. Good vessel wall delineation and blood suppression over the entire arteries were found on all multi-contrast images (Fig. 7). An overview of vessel wall across multiple vascular beds could be provided by 3D curved MPR of multi-contrast images which would be beneficial for plaque distribution analysis and risk assessment (Figs. 8 and 9).

### Image quality

In general, 3D multi-contrast VWI was found to provide good image quality at extracranial segments and adequate image quality at intracranial segment respectively (Table 2). In two patients, the images at extracranial carotid segments were affected by motion artifacts, which adversely influenced with vessel wall delineation. In seven patients, the vessel wall depictions on SNAP images at intracranial segment were found to be unilaterally (6 out of 7) or bilaterally (1 out of 7) inadequate. In other 18 patients, good or adequate images were successfully achieved in both intra- and extracranial segments for each sequence.

**Table 2:** The image quality score on vessel wall delineation for 3D multi-contrast sequences

	3D-MERGE	T2-weighted VISTA	SNAP
Observer 1			
CCA	3.61 ± 0.76	3.39 ± 0.85	3.06 ± 0.76
BULB	3.03 ± 0.56	3.00 ± 0.57	2.67 ± 0.59
ICA C2	2.92 ± 0.49	2.56 ± 0.70	2.78 ± 0.49
ICA C5	3.36 ± 0.61	3.00 ± 0.66	2.86 ± 0.51
MCA M1	2.44 ± 0.38	2.44 ± 0.34	2.33 ± 0.42
Observer 2			
CCA	3.72 ± 0.67	3.42 ± 0.90	3.22 ± 0.96
BULB	3.72 ± 0.57	3.44 ± 0.78	3.22 ± 0.83
ICA C2	3.14 ± 1.08	2.89 ± 0.99	3.50 ± 0.71
ICA C5	3.42 ± 0.86	2.97 ± 0.93	3.08 ± 0.94
MCA M1	2.64 ± 0.95	3.00 ± 0.91	2.86 ± 0.89

### Quantitative measurement

**Fig. 10:**
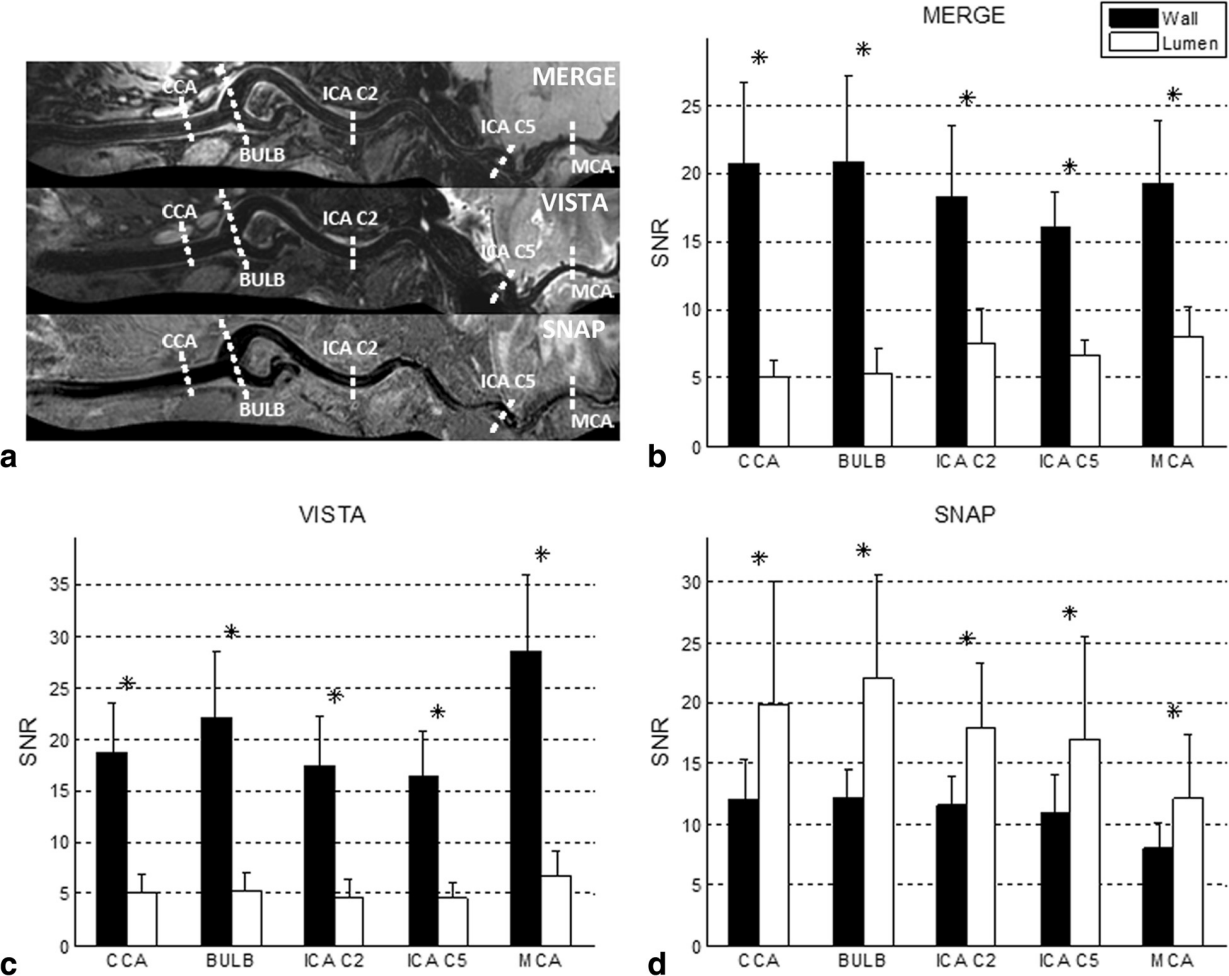
Quantitative SNR measurements of wall and lumen at five locations illustrating the blood suppression effectiveness. The five locations for SNR measurements are denoted by the dashed lines in one MPR result (**a**). (**b** - **d**) show the results of SNR measurement on wall and lumen at different locations for each 3D sequence. Note the lumen signal measured on SNAP images is actually in opposite polarity compared to the wall signal. Therefore the CNR of SNAP is SNR_w_ + SNR_l_ while the CNR of other two sequences are SNR_w_-SNR_l_. Marker "*" indicates the significant difference between wall and lumen signal were found by Wilcoxon signed rank sign test

The averaged SNR_w_ from all five locations generally showed much greater value over the averaged SNR_l_ (3D-MERGE: SNR_w_ = 19.07 ± 6.13, SNR_l_ = 6.56 ± 2.60, P < 0.0001; T2-weighted VISTA: SNR_w_ = 20.64 ± 7.81, SNR_l_ = 5.29 ± 2.26, P < 0.0001; SNAP: SNR_w_ = 10.96 ± 3.51, SNR_l_ = 17.79 ± 9.44, P < 0.0001), indicating that blood suppression can be obtained for each 3D sequence. More specifically, SNR_w_ and SNR_l_ measured at five locations on 3D-MERGE, T2-weighted VISTA and SNAP images were shown in Fig. 10. The blood signal in all multi-contrast images can be suppressed effectively at all five measured locations and CNR_w-l_ calculated at these locations on each sequence were listed in Table 3.

**Table 3:** The Wall-Lumen CNR for 3D multi-contrast sequences

	3D-MERGE	T2-weighted VISTA	SNAP
CCA	15.56 ± 5.20	13.55 ± 4.45	31.86 ± 12.40
BULB	15.62 ± 5.03	16.81 ± 5.14	34.21 ± 9.37
ICA C2	10.83 ± 3.12	12.80 ± 3.56	29.59 ± 6.13
ICA C5	9.39 ± 2.01	11.72 ± 3.46	27.96 ± 10.75
MCA M1	11.24 ± 2.98	21.86 ± 6.15	20.17 ± 5.84

## Discussion

The 3D multi-contrast BB imaging technique can provide a rapid overview for joint intra- and extracranial VWI. The longitudinal coverage is 25 cm, with isotropic spatial resolution of 0.8 mm and a total acquisition of 15 mins. This technique can potentially be used clinically to examine atherosclerotic plaques in both intra- and extracranial vascular beds for screening and/or diagnosis. Comparing to conventional angiographic techniques, this technique is able to provide information of the vessel wall and atherosclerotic plaque in addition to luminal stenosis. Direct visualization of vessel wall and the ability to assess high risk plaques may significantly improve the imaging ability to identify culprit lesions in subjects with stroke and/or TIA, and present clinicians with useful information to decide on the optimal treatment options. This technique may also be used in screening studies to identify subjects with high risk plaques.

The receiving coil plays an important role for joint intra- and extracranial VWI both in its extended longitudinal coverage and improved SNR. The extended longitudinal coverage offers a rapid overview of bilateral arterial vessel walls with a single 3D slab acquisition and the improved SNR contributes to either smaller isotropic voxel size or accelerated imaging. The isotropic dataset can also be beneficial for improving the individual adaptability of wall visualization via curved MPR along vessels. To meet these two major concerns, a recently developed 36-channel coil is adopted in this study with a neurovascular mode to cover the entire intra- and extracranial arteries as well as its dedicated carotid coil elements to provide high SNR imaging.

The 3D multi-contrast information including T1, T2 and heavy T1 weighting has the potential to identify different plaque components similarly to 2D multi-contrast technique [14–16], but can cover more arteries from extracranial carotid to intracranial segments (Figs. 7, 8 and 9). As shown in Fig. 7, the plaque identified at carotid bifurcation with hyperintensity in T2-weighted VISTA and hypointensity in SNAP indicates loose matrix like component, while in Fig. 9 the hyperintense area in SNAP indicated by white arrow demonstrates the IPH component. Moreover, these 3D multi-contrast images offer complementary information on vessel wall delineation. The overall image quality after considering all three multi-contrast images is expected to be further improved compared to the separate qualitative score evaluated in this study.

Effective blood suppression and good or adequate image quality on vessel wall delineation can be achieved for all 3D multi-contrast sequences. Wall SNR is adequately higher over the lumen SNR at all five locations covering bifurcation and major torturous vessels from CCA to MCA for 3D-MERGE and T2-weighted VISTA. Lumen SNR measured in SNAP show larger values than wall SNR but actually they are in contrary polarity. This significant wall lumen difference/CNR reflects the inner wall boundary visibility. However, the wall lumen CNR for each sequence was not consistent across the five locations. The coil sensitivity profile can be another possible common explanation for the signal inhomogeneity of all sequences, especially for those segments from ICA C2 to ICA C5 which are much further away from the surface coil compared to other segments.

For 3D-MERGE, the lumen signal in ICA and MCA segments was found to be relatively larger than the values in CCA and carotid bifurcation segments (P < 0.01). This is because iMSDE preparation module [19] depends on the dephasing of flowing blood spins within each luminal voxel, and the flow pattern in ICA and MCA tends to be more uniform than CCA and bifurcation. For T2-weighted VISTA, the high wall SNR measured in MCA segments (P < 0.01) may be due to CSF contamination within the imaging voxel. VISTA performance in ICA C2, and ICA C5 was better than 3D-MERGE possibly because a spin echo based sequence is not as sensitive to susceptibility at base of skull as a gradient based sequence. For SNAP, the lumen SNR in general showed a decaying trend along the blood flow direction which indicates the blood at distal part experience more than one IRTR cycle especially for large coverage scans leading to reduced blood suppression compared to proximal regions. On the other hand, in some cases, non-inverted blood can flow into CCA segments during the long TI time causing lumen signal polarity error at CCA. This gives rise to the fact that most of lumen signals remain in the positive value of corrected real images. Therefore, lumen SNR measured from negative value of corrected real images in CCA segments shows a smaller mean value and larger standard deviation in Fig. 10(d). This suggests that moving the patient table to ensure inflowing blood inversion before image acquisition can alleviate this problem.

A limitation of large coverage is that the selected sequence parameters may not be optimal for vessel wall delineation in the entire intra- and extracranial vasculatures because the background tissue is different such as muscle in the neck compared to gray matter and white matter in the brain. Nevertheless, the sequence parameters used in this study are available for joint intra- and extracranial VWI with acceptable image quality. Another limitation is potential misregistration among these 3 multi-contrast images due to the sequential imaging scan order. However all three sequences were obtained with identical isotropic voxel dimensions that can allow registration by post-processing. Spatial resolution used in this study might not be optimal for MCA imaging whose vessel caliber is smaller compared to extracranial vessels. Although higher spatial resolution can be achieved at the cost of increased scan duration, 0.8 mm isotropic scan was used in this study to meet the clinical requirement on scan duration while retaining image quality for the majority of the intra- and extracranial arteries. Several parallel imaging and/or compressed sensing methods to further enhance acquisition efficiency have been applied to applications including VWI [34, 35] or sequential multi-contrast imaging [36]. With these acceleration methods, the proposed 3D multi-contrast VWI technique can be further improved regarding the voxel size, imaging FOV and scan duration. In addition, it is noteworthy to point out that further comparison with clinical gold-standard and/or histology is still required for validation of this technique for plaque components characterization.

Three investigated 3D imaging sequences can be used either individually or in combination depending on the specific clinical purpose. 3D-MERGE can provide clear vessel wall depiction in both intra- and extracranial vasculature that is beneficial for the plaque burden measurement. T2-weighted VISTA and SNAP can be regarded as detection tools for high risk plaque components including necrotic core, IPH and calcification. However, it is not recommended to utilize T2-weighted VISTA alone due to the CSF contamination problem at intracranial segment. If contrast injection is permitted for patient scan, post-contrast T1 weighted imaging for example 3D-MERGE [37] can also be incorporated to identify lipid rich necrotic core and fibrous cap.

## Conclusions

A 3D multi-contrast imaging technique at 3 T has been demonstrated for joint intra- and extracranial VWI with isotropic voxels, effective blood suppression, good or adequate image quality and clinically acceptable overall scan time. Its performance on vessel wall delineation and blood suppression has been demonstrated by qualitative image scoring and quantitative lumen/wall SNR measurements in this preliminary patient study. The proposed protocol has the potential to discriminate plaque components by multi-contrast analysis including T1, T2 and heavy T1 weighting. Further clinical verification is required for this promising tool on plaque distribution and plaque components characterization across multiple arteries.
